# Raman Spectroscopy and Imaging for Cancer Diagnosis

**DOI:** 10.1155/2018/8619342

**Published:** 2018-06-07

**Authors:** Sishan Cui, Shuo Zhang, Shuhua Yue

**Affiliations:** ^1^School of Biological Science and Medical Engineering, Beihang University, Beijing 100083, China; ^2^Beijing Advanced Innovation Center for Biomedical Engineering, Beihang University, Beijing 100083, China

## Abstract

Raman scattering has long been used to analyze chemical compositions in biological systems. Owing to its high chemical specificity and noninvasive detection capability, Raman scattering has been widely employed in cancer screening, diagnosis, and intraoperative surgical guidance in the past ten years. In order to overcome the weak signal of spontaneous Raman scattering, coherent Raman scattering and surface-enhanced Raman scattering have been developed and recently applied in the field of cancer research. This review focuses on innovative studies of the use of Raman scattering in cancer diagnosis and their potential to transition from bench to bedside.

## 1. Introduction

**Figure 1:**
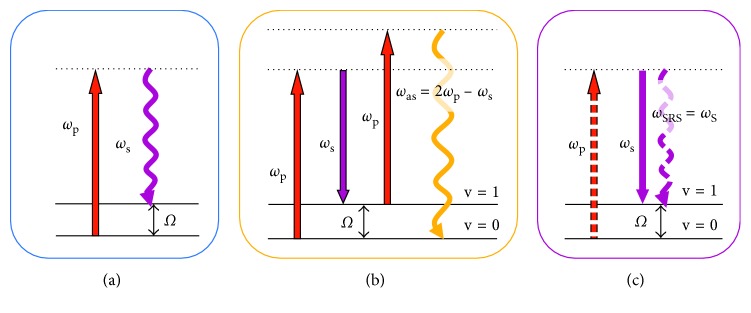
Energy diagrams of spontaneous Raman scattering (a), CARS (b), and SRS (c).

Cancer remains the world's grand challenge. There is an urgent need for development of new techniques for cancer screening, diagnosis, and intraoperative surgical guidance. Raman scattering has long been used to assess chemical compositions in cells and tissues, based on interaction with the vibrational modes of common molecular bonds in the sample. Thus, the alteration of molecular signatures in a cell or tissue undergone disease transformation can be detected by Raman scattering noninvasively without labelling. It is conceivable that Raman spectroscopy is a desirable tool for cancer diagnosis [1–5]. However, due to small cross section (∼10^−30^ cm^2^ per molecule), spontaneous Raman scattering (shown in Figure 1(a)) requires a long integration time, which hinders its biological and medical applications.

In order to enhance the Raman scattering signal level, coherent Raman scattering (CRS) microscopy has been developed [6]. As shown in Figures 1(b) and 1(c), in most CRS imaging experiments, two excitation fields are used, denoted as pump (*ω*_p_) and Stokes (*ω*_s_). When the beating frequency (*ω*_p_−*ω*_s_) matches with a molecular vibration mode, coherent anti-Stokes Raman scattering (CARS) at the frequency of “(*ω*_p_−*ω*_s_) + *ω*_p_” and stimulated Raman scattering (SRS) at the frequency of “*ω*_s_” or “*ω*_p_” will occur simultaneously. Owing to large signal level in CRS microscopy, CRS imaging is ∼1000 times faster than a line-scan Raman microscopy and ∼10,000 times faster than a point-scan Raman microscopy [7]. The advantage of SRS over CARS lies in the fact that the SRS signal is completely free of the nonresonant background, which renders SRS microscopy a highly sensitive and quantitative method for biochemical imaging [8–11]. Besides, SRS can be operated under ambient light. In parallel, rapid advances in nanotechnology have led to the development of surface-enhanced Raman scattering (SERS), which can also tremendously enhance Raman signals although in a labelling manner [12, 13].

With such capabilities, Raman scattering-based techniques can find wide applications in the field of cancer diagnosis. In this review, we summarize the recent developments and applications of Raman scattering-based techniques for cancer diagnosis. In particular, we highlight the innovative studies of three important techniques, spontaneous Raman spectroscopy, CRS, and SERS, and their potential to transition from bench to bedside.

## 2. Spontaneous Raman Scattering for Cancer Diagnosis

### 2.1. *Ex Vivo*

#### 2.1.1. Biofluids

Bhattacharjee et al. employed Raman spectroscopy to diagnose breast cancer using urine in a rat model and obtained classification efficiencies of 80% and 72% by using principal component analysis and principal component-linear discriminant analysis, respectively [14]. In parallel, Elumalai et al. utilized Raman spectroscopy to characterize urine of normal and oral cancer patients and found that principal component analysis-based linear discriminant analysis was able to differentiate normal patients from cancer patients with an accuracy of 93.7%, a sensitivity of 98.6%, and a specificity of 87.1% [15]. Besides, Sahu et al. carried out Raman spectral analysis of serum from oral cancer patients and healthy subjects and found that Raman bands of beta-carotene and DNA content could be used for oral cancer diagnosis [16, 17].

#### 2.1.2. Tissues

*(1) Gastrointestinal Cancer*. Almond et al. evaluated the capability of endoscopic Raman spectroscopy to grade Barrett's esophagus-associated high-grade dysplasia and adenocarcinoma based on the biochemical profile of different tissue types in 673 *ex vivo* esophageal tissue samples from 62 patients and demonstrated a sensitivity of 86% and a specificity of 88% [18]. Hsu et al. were able to differentiate gastrointestinal stromal tumors from gastric adenocarcinomas and normal mucosae using confocal Raman microspectroscopy, based on different Raman signals corresponding to phospholipids and protein structures [19]. The authors further demonstrated that confocal Raman microspectroscopy could be used to differentiate four histological types of gastric adenocarcinomas, including papillary adenocarcinoma, tubular adenocarcinoma, mucinous adenocarcinoma, and signet ring cell adenocarcinoma [20]. Petersen et al. carried out Raman fiber-optical measurements of colon biopsy samples taken during colonoscopy and showed a diagnostic accuracy over 70% [21].

*(2) Skin Cancer*. Nijssen et al. showed that Raman spectroscopy could discriminate basal cell carcinoma from its surrounding tissue in the unstained frozen sections of 15 basal cell carcinoma specimens [22]. Gniadecka et al. were one of the first to investigate the feasibility of Raman spectroscopy in melanoma diagnosis. By neural network analysis of Raman spectra, the authors found structural alterations in proteins and lipids in intact cancer tissues and obtained 85% sensitivity and 99% specificity for melanoma diagnosis [23]. Bodanese et al. employed Raman spectroscopy to identify malignant basal cell carcinoma and melanoma *in vitro*, with proteins, lipids, and melanin accounting for 95.4% of all spectral variation [24]. The authors further showed higher sensitivity and specificity for the principal component analysis model compared to biochemical models [25]. Because construction of fiber-optic probes suitable for Raman spectroscopy is complicated in the fingerprint region, Nijssen et al. evaluated and confirmed that the high-wavenumber region also provided sufficient information for accurate diagnosis of basal cell carcinoma [26].

*(3) Breast Cancer*. Frank et al. [27] conducted one of the first studies of Raman spectroscopy for breast cancer diagnosis. Spectra revealed Raman differential features of lipids and carotenoids in normal and cancerous biopsies. In a later study, the authors found much weaker lipid bands and more prominent collagen bands in diseased specimens compared to their normal counterparts [28]. By applying Raman spectroscopy and principal component analysis, Haka et al. found that type II microcalcifications formed in malignant ducts typically contain a smaller amount of calcium carbonate and a larger amount of protein than those formed in benign ducts [29]. Three years later, the authors used Raman spectroscopy to analyze benign and malignant lesions in human breast tissues collected from 58 patients. By combining nine representative spectra for the morphological and chemical features of breast tissue, a linear combination model was developed for distinguishing cancerous tissues from normal and benign tissues attaining 94% sensitivity and 96% specificity, with fat and collagen as the key parameters in the diagnostic algorithm [30].

*(4) Lung Cancer*. Huang et al. conducted near-infrared (NIR) Raman spectroscopy of tissue specimens collected from patients and found that the ratio of Raman intensities at 1,445 to 1,655 cm^−1^ could be used to identify malignant bronchial tissue [31]. Magee et al. designed a mini-fiber-optic Raman probe, which was suitable for insertion into the working channel of a bronchoscope, and accurately classified normal and malignant lung tissues *ex vivo* [32].

*(5) Brain Cancer*. Koljenovic et al. demonstrated the capability of Raman spectroscopy to discriminate vital tumor from necrotic tissue in unfixed cryosections of glioblastoma collected from 20 patients [33]. Five years later, the authors further analyzed brain tissue slices from 7 pigs by using high-wavenumber Raman spectroscopy in a single fiber-optic probe setup, showing the potential of Raman spectroscopy as an intraoperative guidance tool [34]. Krafft et al. applied the spectral unmixing algorithm to identify cell density and cell nuclei in Raman images of primary brain tumor tissue sections. This work showed that morphology and composition correlated well with histopathology and provided complementary information for better diagnosis [35].

### 2.2. *In Vivo*

#### 2.2.1. Animal Models

Kirsch et al. proved that Raman spectroscopy could be used to detect intracerebral tumors *in vivo* by brain surface mapping with an accuracy of roughly 250 *μ*m [36]. At the same year, Beljebbar et al. demonstrated that Raman spectroscopy could distinguish between normal brain and tumor tissues with 100% accuracy in C6 glioblastoma animal model, based on the biochemical information, primarily the variations in the lipid signals [37].

#### 2.2.2. Studies in Humans

*(1) Gastrointestinal Cancer*. In recent years, the Huang group has made significant contributions to push the use of Raman spectroscopy in gastrointestinal cancer diagnosis *in vivo* during clinical endoscopic examination [38–47]. Huang et al. developed a narrow-band image-guided Raman endoscopy technique for diagnosis of gastric dysplasia *in vivo* [39]. Significant differences in Raman spectra between normal and dysplastic gastric tissues led to a diagnostic sensitivity of 94.4% and a specificity of 96.3%. The albumin, nucleic acid, phospholipids, and histones were found to be the most significant features to construct the diagnostic model [38]. Bergholt et al. integrated novel fiber-optic Raman spectroscopy with semiquantitative spectral modelling and revealed that the biochemical constituents in gastric tissue progressively changed during preneoplastic and neoplastic transformation. A total of 1277 *in vivo* Raman spectra from 83 gastric patients were collected, and a sensitivity of 83.33% and a specificity of 95.80% were obtained for dysplasia and a sensitivity of 84.91% and a specificity of 95.57% were obtained for adenocarcinoma [40, 41]. The authors further characterized *in vivo* Raman spectroscopic features for normal versus cancerous colorectal tissues and showed that partial least squares-discriminant analysis yielded a diagnostic accuracy of 88.8% for colorectal cancer detection [48]. In parallel, Shim et al. also demonstrated feasibility of NIR Raman spectroscopy used during clinical gastrointestinal endoscopy [49].

*(2) Breast Cancer*. Haka et al. demonstrated *in vivo* Raman spectroscopy for margin assessment during partial mastectomy breast surgery in nine patients. Application of their previous diagnostic algorithm led to great sensitivity and specificity for differentiation of normal and cancerous tissues [50]. Brozek-Pluska et al. applied Raman spectroscopy to examine noncancerous and cancerous human breast tissues of the same patient and found that the most significant differences between noncancerous and cancerous tissues were related to carotenoids, proteins, and lipids, especially the unsaturated fatty acids [51].

**Figure 2:**
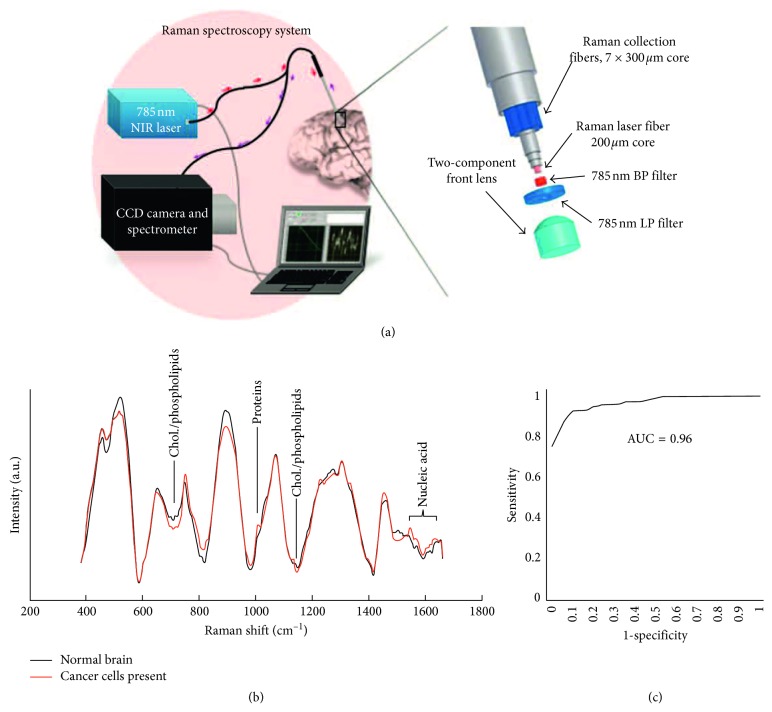
Head-held contact Raman spectroscopy probe technique for live, local detection of cancer cells in the human brain. (a) System setup; (b) Raman spectra of normal and cancerous cells in human brain; (c) diagnostic sensitivity and specificity. Reprinted with permission from Ref. [52].

*(3) Brain Cancer*. Desroches et al. conducted a detailed characterization of handheld Raman spectroscopy system in order to maximize the volume of resected cancer tissue in glioma surgery. Preliminary measurements of normal, necrotic, and cancerous tissues collected from 10 patients demonstrated that necrosis could be distinguished from vital tissue, including normal and cancerous brain tissue, with an accuracy of 87% [52]. In the same year, Jermyn et al. developed a head-held contact Raman spectroscopy probe technique for live, local detection of cancer cells in the human brain [53] (Figure 2). By using this technique, the authors were able to precisely identify cancer cells with 93% sensitivity and 91% specificity.

*(4) Skin Cancer*. Lui et al. evaluated the real-time Raman spectroscopy system for diagnosis of skin cancer *in vivo*. A total of 518 benign and malignant lesions from 453 patients were measured at one second per lesion. Benign, precancer, nonmelanoma, and melanoma lesions were differentiated with sensitivities ranging from 95% to 99% and specificities ranging from 15% to 54% [54].

*(5) Cervical Cancer*. The Huang group has conducted several studies on cervical precancer detection by using Raman spectroscopy *in vivo* [55, 56]. The authors demonstrated that integration of NIR Raman spectroscopy with genetic algorithm-partial least squares-discriminant analysis could identify seven diagnostically significant Raman bands related to proteins, nucleic acids, and lipids and obtained an accuracy of 82.9% for precancer detection [55]. Later on, the authors showed that NIR confocal Raman spectroscopy could further improve the diagnostic accuracy with higher sensitivity and specificity [56]. By analyzing *in vivo* Raman spectra from 93 subjects under clinical supervision, Shaikh et al. revealed abundant collagen in normal cervix and prominent DNA in tumors. Principal component-linear discriminant analysis yielded 97% efficiency to differentiate normal and tumor groups [57].

## 3. Coherent Raman Scattering for Cancer Diagnosis

### 3.1. *Ex Vivo*

#### 3.1.1. Brain Cancer

The Xie group has made tremendous contributions to the development of novel coherent Raman scattering microscopy for neuropathological diagnosis [58–61]. Evans et al. demonstrated the feasibility of CARS microscopy to identify normal brain structures and primary glioma in fresh unfixed and unstained *ex vivo* brain tissues [58]. Five years later, Freudiger et al. developed multicolored coherent Raman imaging to visualize signals of lipids and protein originated from CH_2_ and CH_3_ vibrations in fresh brain tissues. These multicolor coherent Raman images showed almost identical morphological information compared with the corresponding histopathological images [59]. Uckermann et al. performed CARS imaging of brain tissues in an orthotopic mouse model and human glioblastoma at the C-H molecular vibration region. Based on the lipid content, the authors were able to delineate tumor margins and infiltrations with cellular resolution [60]. More recently, Ji et al. employed SRS microscopy to study human brain tumor infiltration in fresh, unprocessed surgical specimens from 22 neurosurgical patients. This study revealed that SRS was capable of detecting tumor infiltration in high agreement with H&E staining. The authors further created a classifier by quantitatively analyzing cellularity, axonal density, and protein : lipid ratio and gained 97.5% sensitivity and 98.5% specificity for detection of tumor infiltration [61].

#### 3.1.2. Lung Cancer

The Wong group developed CARS microscopy for differentiation of lung cancer from nonneoplastic lung tissues based on a prior knowledge including the established pathological workup and diagnostic cellular. A total of 92 fresh frozen lung tissue samples were analyzed, and 91% sensitivity and 92% specificity were obtained for lung cancer diagnosis [62, 63]. By combining deep learning and CARS imaging, the Wong group achieved automated differential diagnosis of lung cancer [64].

### 3.2. *In Vivo*

#### 3.2.1. Animal Model

**Figure 3:**
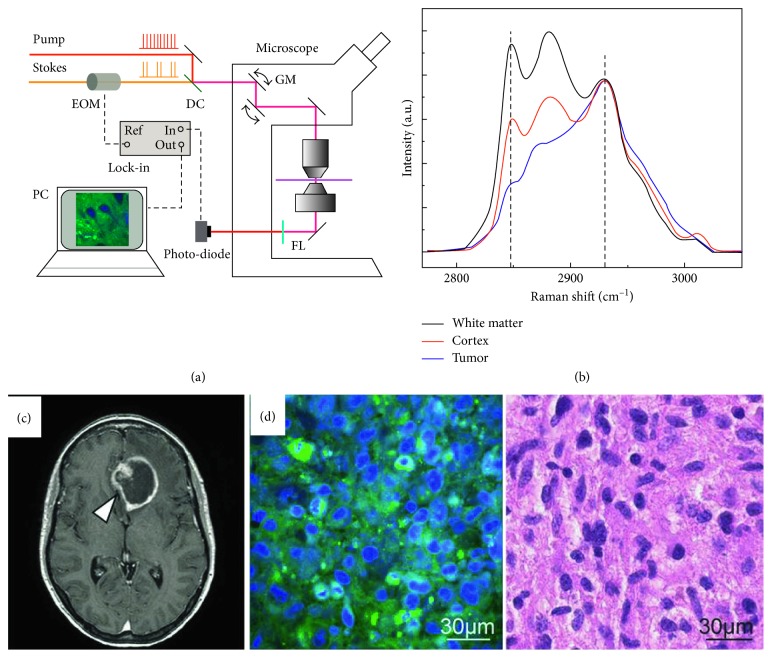
SRS microscopy delineating glioma infiltration in animal models. (a) System setup; (b) Raman spectra of white matter, cortex, and tumor; (c) MRI image of a mouse brain; (d) SRS and the corresponding H&E image of a glioma tissue. Reprinted with permission from Ref. [65].

Ji et al. recently demonstrated the ability of SRS microscopy to delineate glioma infiltration in animal models based on histoarchitectural and biochemical differences. Their results were confirmed by a good correlation between SRS and hematoxylin and eosin microscopy for detection of glioma infiltration (kappa = 0.98). The authors further applied SRS microscopy *in vivo* during surgery to identify tumor margins [65] (Figure 3). Later on, the Ji group developed dual-phase SRS microscopy for real-time two-color imaging, which could reach the maximum speed as in single-color SRS. The authors also proved that this method could perform accurate real-time histology *in vivo* in both transmission and epi modes [66].

#### 3.2.2. Studies in Humans

More recently, Orringer et al. demonstrated a fiber-laser-based SRS microscopy, which could perform rapid intraoperative histology of unprocessed surgical specimens from 101 neurosurgical patients. Quantitatively, the authors found a remarkable concordance of SRS and conventional histology for predicting diagnosis (*κ* > 0.89), with accuracy over 90% [67]. Hollon et al. further utilized this method for the intraoperative diagnosis of pediatric brain tumors, which achieved near-perfect diagnostic concordance (*κ* > 0.90) and an accuracy of 92–96% [68].

## 4. Surface-Enhanced Raman Scattering (SERS) for Cancer Diagnosis

### 4.1. *Ex Vivo*

Dai et al. performed SERS to differentiate human oral cancer cells from normal fibroblast cells *in vitro*, based on the characteristic Raman signal of adenine at 735 cm^−1^ [69]. Grubisha et al. developed an immunoassay based on SERS and achieved a fast femtomolar detection of prostate-specific antigen for prostate cancer screening [70]. Li et al. applied SERS and support vector machine techniques to analyze serum samples from 93 prostate cancer patients and 68 healthy volunteers. A diagnostic accuracy of 98.1% was achieved [71]. Del Mistro et al. conducted a preliminary study on prostate cancer detection by SERS spectroscopy of urine. By using principal component analysis and linear discriminant analysis, this study reached a sensitivity of 100%, a specificity of 89%, and an overall diagnostic accuracy of 95% [72]. Feng et al. used SERS spectroscopy to analyze purified whole proteins from human saliva and achieved an accuracy of 90.2% for nasopharyngeal cancer detection [73]. The authors further showed SERS was able to differentiate healthy subjects, benign breast tumor patients, and malignant breast tumor patients, with >70% sensitivity and >80% specificity, respectively [74].

### 4.2. *In Vivo*

#### 4.2.1. Animal Model

**Figure 4:**
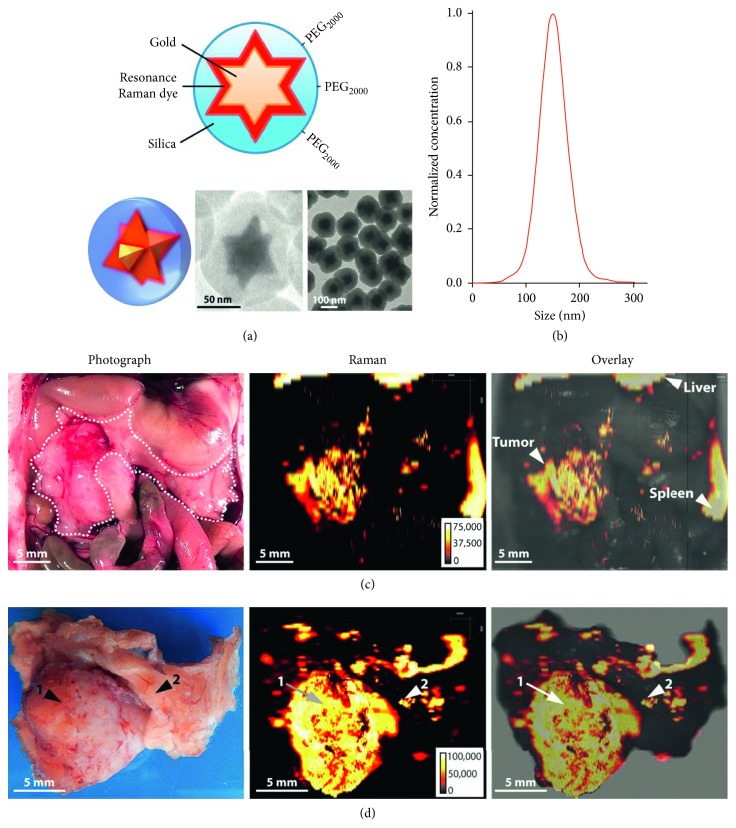
A new generation of SERS nanoparticles used to visualize tumor margins, tumor invasion, and locoregional tumor spread with high precision. (a) Structure of the nanoparticle; (b) size distribution of the nanoparticles; (c) in situ photograph of the exposed upper abdomen in a mouse with a pancreatic cancer in the head of the pancreas (outlined with white dotted line). Corresponding Raman image showing SERRS-nanostar signal in the macroscopically visible tumor in the head as well as small scattered foci of SERRS-signal in other normal-appearing regions of the pancreas. (d) Photographic and high-resolution Raman images of the excised pancreas from (c). Reprinted with permission from Ref. [78].

Mohs et al. integrated NIR contrast agents with a handheld spectroscopic pen device to perform SERS analysis of breast tumor-bearing mice and could identify tumor borders preoperatively and intraoperatively with high accuracy [75]. Dinish et al. intratumorally injected antibody-conjugated SERS nanotags to specifically target three intrinsic cancer biomarkers—EGFR, CD44, and TGF-beta RII in a breast cancer model. SERS signal was specifically detected in tumor-bearing animal with a maximum at 6 hours postinjection [76]. Karabeber et al. developed a SERS nanoparticle-guided handheld Raman scanner to identify tumor tissues in a genetically engineered RCAS/tv-a glioblastoma mouse model. The detection accuracy of this method was more accurate than white light visualization alone [77]. Harmsen et al. developed a new generation of SERS nanoparticles, which could be used to visualize tumor margins, tumor invasion, and locoregional tumor spread with high precision, in mouse models of pancreatic cancer, breast cancer, prostate cancer, and sarcoma [78] (Figure 4).

#### 4.2.2. Studies in Human

Garai et al. have recently developed a miniature, noncontact, optoelectromechanical Raman device attaching to clinical endoscopes and demonstrated that this device could improve accuracy and speed of gastrointestinal cancer diagnosis by using a series of SERS nanoparticles [79].

## 5. Integration of Raman-Based Technologies with Other Optical Modalities for Cancer Diagnosis

### 5.1. *Ex Vivo*

The Popp group integrated Raman spectroscopy, CARS, second harmonic generation (SHG), and two-photon-excited fluorescence (TPEF) imaging on the same platform and obtained multimodal images with distinct features of basal cell and squamous cell carcinoma [80, 81]. The group further utilized multimodal nonlinear imaging method to image brain tissues *ex vivo* and identified cytological and architectural features for tumor grading [82].

### 5.2. *In Vivo*

**Figure 5:**
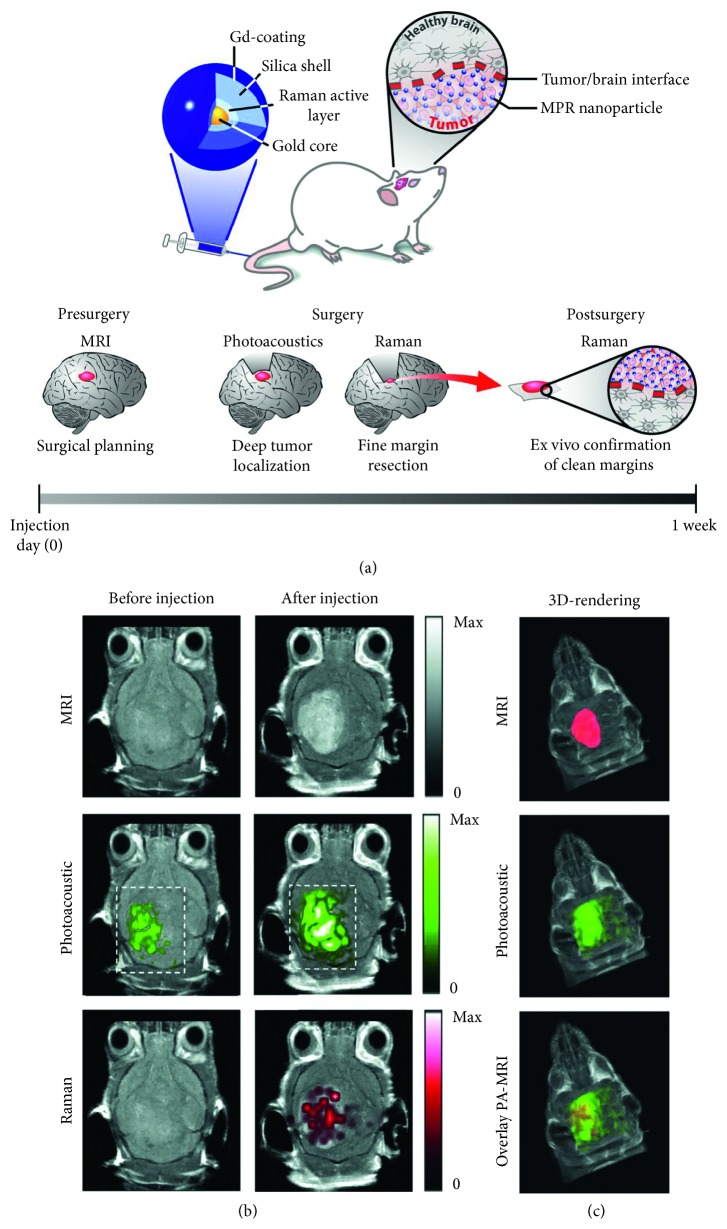
A unique triple-modality magnetic resonance imaging-photoacoustic imaging-Raman imaging (MPR) nanoparticle for selective multimodal imaging of tumor margins in glioblastoma-bearing mice. (a) Triple-modality MPR concept; (b, c) three weeks after orthotopic inoculation, tumor-bearing mice (*n* = 4) were injected intravenously with MPRs (16 nm, 170 *μ*l). Photoacoustic, Raman, and MR images of the brain (skin and skull intact) were acquired before and 2 h, 3 h, and 4 h after injection, respectively. (b) 2D axial MRI, photoacoustic, and Raman images. The postinjection images of all three modalities demonstrated clear tumor visualization. The photoacoustic and Raman images were coregistered with the MR image, demonstrating good colocalization between the three modalities. (c) 3D rendering of MR images with the tumor segmented (red; top); overlay of 3D photoacoustic images (green) over MRI (middle); and overlay of MRI, segmented tumor, and photoacoustic image (bottom) showing good colocalization of the photoacoustic signal with the tumor. Reprinted with permission from Ref. [83].

Kircher et al. formulated a unique triple-modality magnetic resonance imaging-photoacoustic imaging-Raman imaging nanoparticle for selective multimodal imaging of tumor margins in glioblastoma-bearing mice [83] (Figure 5). Jeong et al. developed a dual-modal fluorescence-Raman endomicroscopic system that combined fluorescence and SERS nanoprobes. This system was utilized to simultaneously detect two biomarkers, human epidermal growth factor receptor 2 and epidermal growth factor receptor, in a breast cancer orthotopic model [84]. Kim et al. further demonstrated the capability of the fluorescence-Raman endomicroscopic system for colorectal cancer diagnosis in an orthotopical xenograft model [85]. Lin et al. developed an integrated 4-modality endoscopy system combining white light imaging, autofluorescence imaging, diffuse reflectance spectroscopy, and Raman spectroscopy technologies for *in vivo* endoscopic nasopharyngeal cancer detection [86].

## 6. Conclusions

With the capability of label-free and highly sensitive analysis of biomolecules in situ, Raman scattering-based techniques offer robust tools for cancer diagnosis. Using fiber-optic-based light delivery and collection, Raman scattering-based techniques are mostly performed on accessible tissue surfaces, for example, on the skin, in gastrointestinal tract, or intraoperatively. The strength of Raman scattering lies in the high sensitivity and specificity, which leads to fast and accurate differentiation between malignant or premalignant from normal tissues. The challenge of cancer diagnosis using Raman spectroscopy would still be how to find very specific molecular marker for different types of human cancers. Hyperspectral SRS microscopy, which can quantitatively map different species of molecules, is a good way to discover new molecular markers for cancer diagnosis.

Looking into the future, we would predict three promising directions. One is the rapid histology based on two-color SRS microscopy which can be used in operation room during cancer surgery. The second is in situ molecule-based diagnosis using handheld fast Raman imaging techniques, for example, handheld Raman spectroscopy or hyperspectral SRS microscopy. The third is multimodal imaging and spectroscopy system that integrate advantages of each modality and may offer better diagnosis for cancer.
